# Atopic dermatitis

**DOI:** 10.1186/s13223-024-00927-2

**Published:** 2024-12-09

**Authors:** Stuart Carr, Rebecca Pratt, Fred White, Wade Watson

**Affiliations:** 1 Snö Asthma & Allergy, Abu Dhabi, United Arab Emirates; 2 Division of Allergy and Immunology, McMaster University, Hamilton, Ontario, Aviva Medical Specialist Clinic, St. Catharines, Ontario Canada; 3 Division of Allergy and Immunology, Western University, London, Ontario Canada; 4 Division of Allergy, IWK Health Centre, Department of Pediatrics, Dalhousie University, Halifax, Nova Scotia Canada

**Keywords:** Atopic dermatitis, Diagnosis and management, Emollients, Skin care practices, Topical corticosteroids, Topical calcineurin inhibitors, Phosphodiesterase-4 inhibitors, Biologics, JAK inhibitors

## Abstract

Atopic dermatitis (AD) is a common, chronic skin disorder that can significantly impact the quality of life (QoL) of affected individuals as well as their families. Although the pathogenesis of the disorder is not yet completely understood, it appears to result from the complex interplay between defects in skin barrier function, environmental and infectious agents, and immune dysregulation. There are no diagnostic tests for AD; therefore, the diagnosis is based on specific clinical criteria that take into account the patient’s history and clinical manifestations. Successful management of the disorder requires a multifaceted approach that involves education, optimal skin care practices, anti-inflammatory treatment with topical corticosteroids, topical calcineurin inhibitors (TCIs) and/or phosphodiesterase-4 (PDE-4) inhibitors, the management of pruritus, and the treatment of skin infections. Systemic immunosuppressive agents may also be used, but are generally reserved for severe flare-ups or more difficult-to-control disease. Newer systemic agents, such as Janus Kinase (JAK) inhibitors and biologics, have a more favourable safety and efficacy profile than the older, traditional systemic immunosuppressives. Topical corticosteroids are the first-line pharmacologic treatments for AD, and evidence suggests that these agents may also be beneficial for the prophylaxis of disease flare-ups. Although the prognosis for patients with AD is generally favourable, those patients with severe, widespread disease and concomitant atopic conditions, such as asthma and allergic rhinitis, are likely to experience poorer outcomes. Newer systemic agents have been approved which are greatly improving the QoL of these patients.

## Key take-home messages

• AD is the most common skin disorder in children, and significantly impacts QoL.
• The diagnosis of AD is based on specific diagnostic criteria that take into account the patient’s history and clinical manifestations.
• Random or screening allergy tests to foods are not recommended. Allergy testing using SPTs or serum-specific IgE measurements may be useful for identifying triggers of AD if the patient’s history is suggestive of allergies to foods or other environmental factors.
• Early, consistent application of emollients may help prevent AD in infants at increased risk.
• Optimal skin care practices and topical corticosteroids are the mainstay of therapy for AD.
• TCIs and PDE-4 inhibitors are a second-line alternative to topical corticosteroids.
• The skin of most patients with AD is heavily colonized with *S. aureus*; therefore, topical and/or systemic antibiotic therapy may be required for overt infections.
• Specialist referral may be helpful for severe flare-ups or more difficult-to-control disease. In these cases, biologics or systemic immunosuppressive agents, including the selective JAK inhibitors, may be utilized which have significant beneficial effects on QoL and eczema severity.
• Referral to an allergist should also be considered for those patients or families worried about the potential role of allergies and for those who also have significant allergic co-morbidities, such as food allergy or asthma.

## Introduction

Atopic dermatitis (AD) is a chronic, highly pruritic (itchy) inflammatory skin disease, and is one of the most common skin disorders in children [1]. The disorder results in significant morbidity and adversely affects quality of life (QoL) [2]. Not only are patients affected by the social stigma of a visible skin condition, but the intense itching characteristic of the disease often leads to skin trauma and significant sleep disturbances. This, in turn, can have a significant adverse impact on mental health, with both depression and anxiety, as well as attention-deficit/hyperactivity disorder (which is speculated to be a consequence of sleep disturbances), observed in people with AD [3, 4]. In addition, management of the condition necessitates the frequent application of emollients (agents that soothe, moisturize, and soften the skin) and topical medications, as well as physician visits. AD also poses a significant economic burden with an estimated annual cost in Canada of $1.4 billion [5]. A recent systematic review estimated the average annual direct cost of AD to be $4411 USD per patient, with additional indirect costs of $9068 USD per patient [3].

Current evidence suggests that AD is a primary skin barrier defect that facilitates the development of other atopic conditions [6, 7]. In fact, AD is often the initial step in the “atopic march” (the sequential development of allergic disease manifestations during early childhood), which leads to asthma and/or allergic rhinitis in the majority of afflicted patients [8]. Early AD may also be a causative factor in the development of food allergy [9], with cutaneous exposure to food allergens appearing to be an important route by which infants sensitize, especially in those with more severe AD [10].

Newer insights into AD suggest that both structural abnormalities of the skin and immune dysregulation play important roles in the pathophysiology of the disease. Therefore, optimal management of AD requires a multifaceted approach aimed at healing and protecting the skin barrier and addressing the complex immunopathogenesis of the disease [11, 12]. This article provides an overview of current literature related to the epidemiology, pathophysiology, diagnosis, and appropriate management of AD.

## Pathophysiology

The pathogenesis of AD is complex, multifactorial, and still evolving, and a comprehensive review of this topic is beyond the scope of this article. In brief, the disorder appears to result from the interaction between defects in skin barrier function, immune dysregulation, and environmental and infectious agents [6, 7, 13, 14]. Skin barrier abnormalities are often associated with mutations within, or impaired expression of, the filaggrin gene (*FLG*), which encodes a structural protein essential for skin barrier formation, although genome-wide analyses have now identified at least 30 different susceptibility regions for AD [15, 16]. The skin of individuals with AD has also been shown to be deficient in ceramides (lipid molecules) as well as antimicrobial peptides such as cathelicidins, which represent the first-line of defense against many infectious agents. These skin barrier abnormalities lead to transepidermal water loss (passage of water from inside the body through the epidermal layer of the skin to the surrounding atmosphere) and increased penetration of allergens and microbes into the skin. The infectious agents most often involved in AD are *Staphylococcus aureus* (*S. aureus*), which colonizes in approximately 90% of AD patients, herpes simplex viruses, and *molluscum contagiosum virus* [17]. Defective innate immune responses contribute to increased bacterial and viral infections in patients with AD. This interplay of factors, including the increasingly-recognized roles of innate lymphoid cells (ILCs) [18] and Janus Kinase (JAK) signaling [19], leads to T-cell responses in the skin (initially a predominantly type 2 [T2] inflammatory response and, later, a predominantly type 1 [T1] response) with resultant release of chemokines and proinflammatory cytokines (e.g., interleukin [IL]-4, IL-5 and tumour necrosis factor) that promote immunoglobulin E (IgE) production and systemic inflammatory responses, leading to pruritic inflammation of the skin.

## Epidemiology

The prevalence of AD has increased over the past 30 years. It is currently estimated that 15–20% of children, and up to 10% of adults, around the globe are affected by the disorder [20]. AD often starts in early infancy; approximately 45% of all cases begin within the first 6 months of life, 60% during the first year, and 85% before 5 years of age. In fact, many neonates destined to develop AD already have measurably increased transepidermal water loss on their second day of life [21], and this finding is strongly predictive of future food allergy [22]. Fortunately, only around 10% of children with AD have severe disease [23], and up to 70% of affected children will go into clinical remission before adolescence [24, 25].

As mentioned earlier, children with AD are at increased risk for developing food allergies, asthma and allergic rhinitis, and these risks are further increased depending on the severity, age of onset, and duration of AD [26, 27]. As many as 40% of children with moderate-to-severe AD will develop IgE-mediated food allergies [28–30], and severe AD in infancy is a major risk factor for allergies to egg and peanut specifically [9, 22, 31]. A systematic review suggested that AD of increased severity and chronicity is particularly associated with food allergy, and that AD precedes the development of food allergy, indicating a possible causal relationship [9]. Evidence also suggests that of those who develop AD before the age of 2, 50% will develop asthma during subsequent years [32]. Furthermore, those children with AD who develop asthma and allergic rhinitis are more likely to have severe disease [32].

## Diagnosis

There are no specific diagnostic tests for AD. Diagnosis of the disorder is based on specific criteria that take into account the patient’s history and clinical manifestations. Although various diagnostic criteria for AD have been proposed and validated, the application of many of these criteria is time consuming and often necessitates invasive testing. Table 1 provides simplified criteria that are easy to use, do not require invasive testing, and have been shown to have a high sensitivity and specificity for the diagnosis of AD [33–36]. Using these criteria, the diagnosis of AD requires the presence of an itchy skin condition (or parental/caregiver reports of scratching or rubbing in a child) plus three or more minor criteria, which vary depending on the patient’s age.

**Table 1:** Diagnostic criteria for AD [33–35]

**Major criteria:**
Patient must have: · An itchy skin condition (or parental/caregiver report of scratching or rubbing in a child)
**Minor criteria**:
Plus three or more of the following minor criteria: *Older children/adults*: · History of itchiness in skin creases (e.g., folds of elbows, behind the knees, front of ankles, around the neck) · Personal history of asthma or allergic rhinitis · Personal history of general dry skin in the last year · Visible flexural dermatitis (i.e., in the bends or folds of the skin at the elbow, knees, wrists, etc.) · Onset under age 2 years Children < 4 years: * · History of itching of the cheeks · History of atopic disease in a first-degree relative · Eczema of cheeks, forehead and outer limbs

* Early onset not always diagnostic in children under 4 years of age

**Fig. 1:**
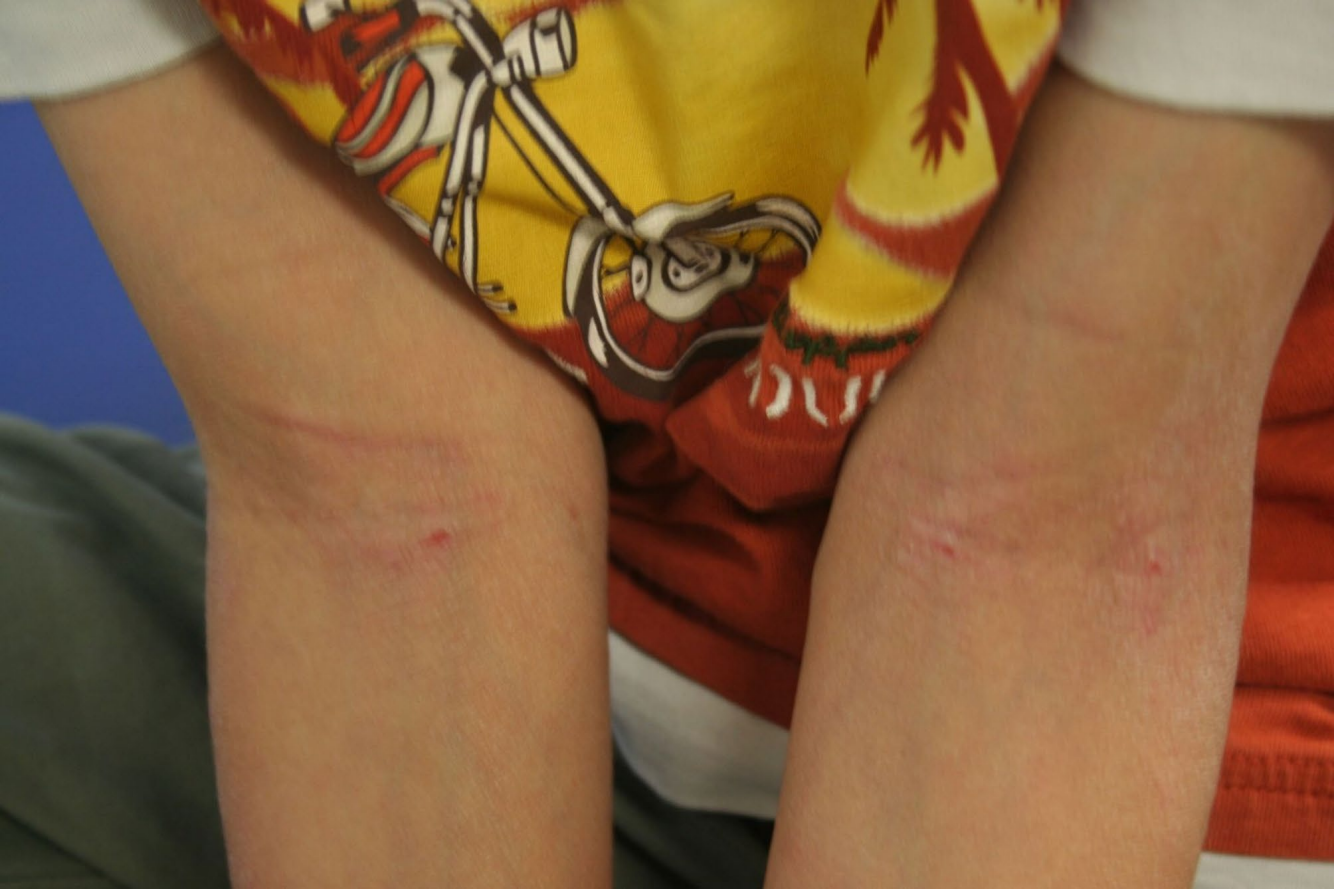
AD of the flexural surfaces of the extremities

**Fig. 2:**
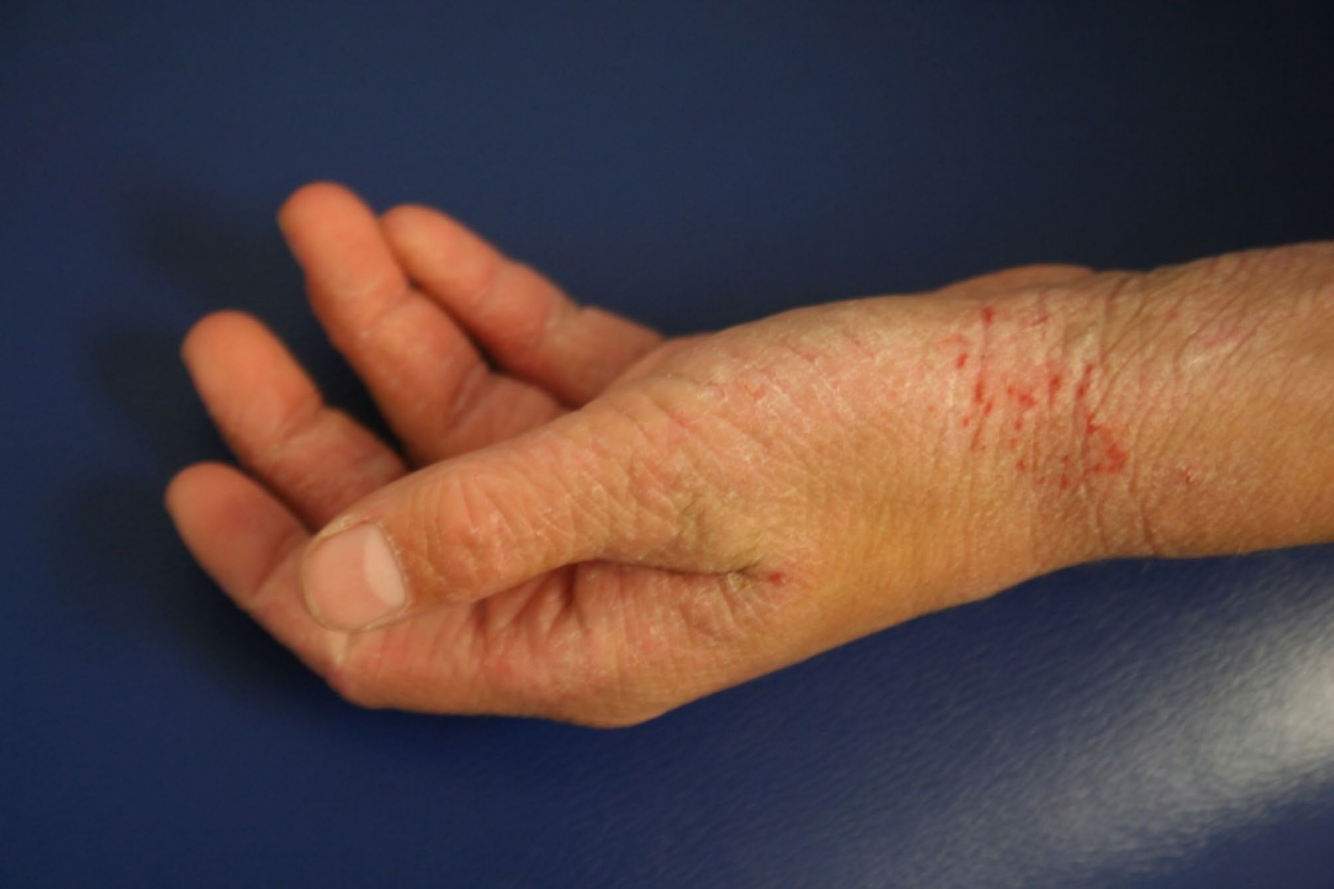
AD of the hands

**Fig. 3:**
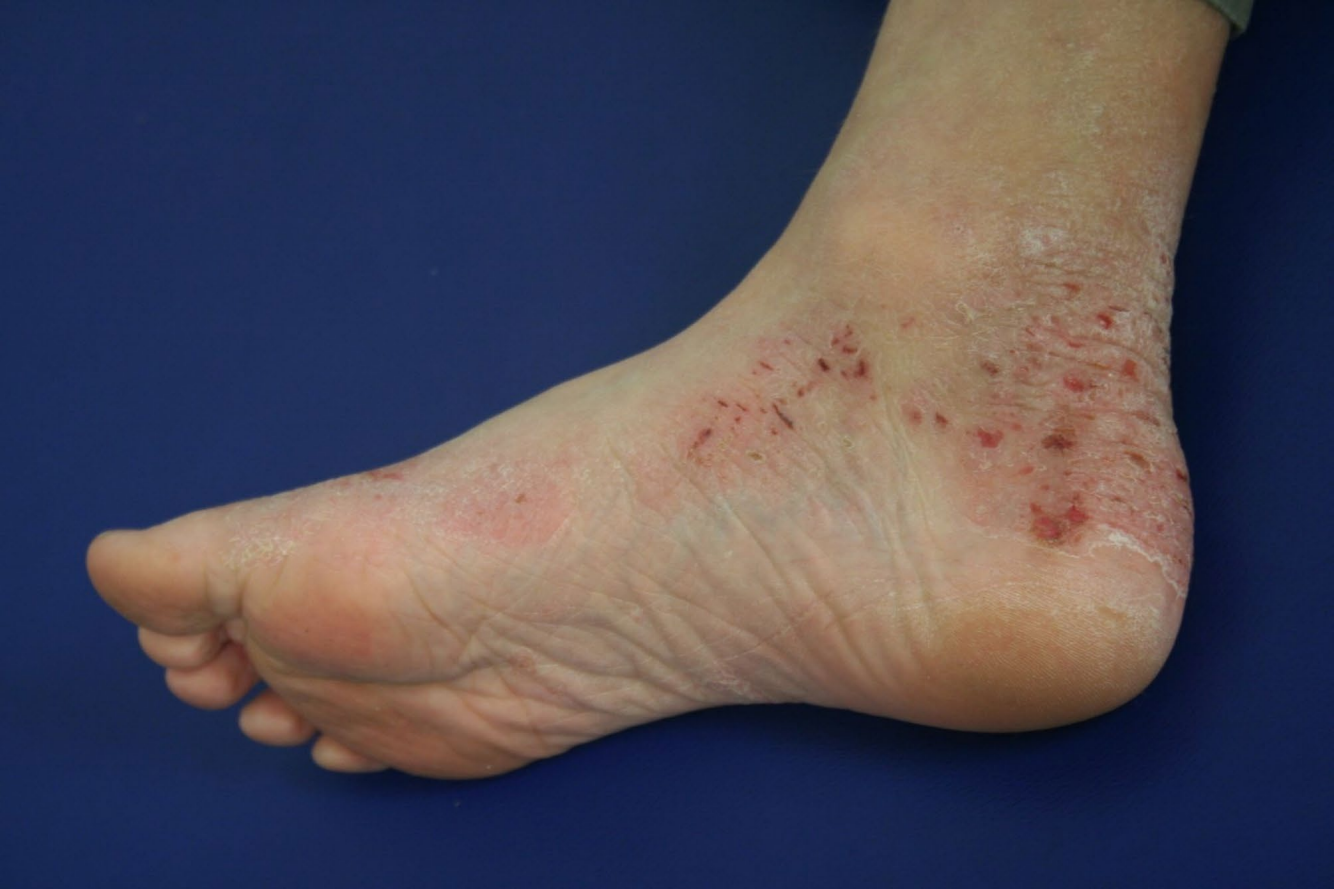
AD of feet

The clinical manifestations of AD vary with age (see Table 2). In infants, the scalp, face, neck, trunk and extensor (outer) surfaces of the extremities are generally affected, while the diaper area is usually spared. Children typically have involvement of the flexural surfaces of the extremities (i.e., fold/bend at the elbow and back of the knee), neck, wrists and ankles (see Fig. 1). In adolescence and adulthood, the flexural surfaces of the extremities, hands (see Fig. 2) and feet (see Fig. 3) are usually affected. Regardless of age, the itching associated with AD generally continues throughout the day and worsens at night, leading to sleep loss and substantial impairments in QoL [2, 12].

**Table 2:** Clinical manifestations of AD

**Infants** **(0**–**2 years)** · Extensor surfaces of extremities · Face (forehead, cheeks, chin) · Neck · Scalp · Trunk
**Childhood (2 years to puberty)** · Flexural surfaces of extremities · Neck · Wrists, ankles
**Adolescence/adulthood** · Flexural surfaces of extremities · Hands, feet

It is sometimes difficult to differentiate AD from other skin conditions (e.g., seborrheic dermatitis, contact dermatitis, psoriasis, scabies); however, a family history of atopy and the distribution of lesions are helpful in making the diagnosis in many cases. Psoriasis, for example, usually affects the extensor rather than flexural surfaces, and often involves the fingernails, palms of the hands and soles of the feet. Seborrheic dermatitis typically involves the diaper area and scalp in infants, and the face in adults (e.g., sides of the nose, eyebrows, external ear canal). Furthermore, unlike AD, a family history of atopic disease is less common in patients with seborrheic or contact dermatitis. Scabies is generally associated with the presence of pustules on the palms, soles, genitalia and between the fingers. Other conditions that need to be considered in the differential diagnosis of AD are nutritional deficiencies, malignancies, as well as keratinization disorders and immunodeficiency disorders that are associated with skin manifestations (see Table 3) [12].

**Table 3:** Common differential diagnosis of AD [12]

	Main age group affected	Frequency*	Characteristics and clinical features
**Other types of dermatitis**			
Seborrheic dermatitis	Infants	Common	Salmon-red greasy scaly lesions, often on the scalp (cradle cap) and napkin area; generally presents in the first 6 weeks of life; typically clears within weeks
	Adults	Common	Erythematous patches with yellow, white, or grayish scales in seborrheic areas, particularly the scalp, central face, and anterior chest
Nummular dermatitis	Children and adults	Common	Coin-shaped scaly patches, mostly on legs and buttocks; usually no itch
Irritant contact dermatitis	Children and adults	Common	Acute to chronic eczematous lesions, mostly confined to the site of exposure; history of locally applied irritants is a risk factor; might coexist with AD
Allergic contact dermatitis	Children and adults	Common	Eczematous rash with maximum expression at sites of direct exposure but might spread; history of locally applied irritants is a risk factor; might coexist with AD
Lichen simplex chronicus	Adults	Uncommon	One or more localised circumscribed lichenified plaques that result from repetitive scratching or rubbing because of intense itch
Asteatotic eczema	Adults	Common	Scaly, fissured patches of dermatitis overlying dry skin, most often on lower legs
**Infectious skin diseases**			
Dermatophyte infection	Children and adults	Common	One or more demarcated scaly plaques with central clearing and slightly raised reddened edge; variable itch
Impetigo	Children	Common	Demarcated erythematous patches with blisters or honey-yellow crusting
Scabies	Children	Common†	Itchy superficial burrows and pustules on palms and soles, between fingers, and on genitalia; might produce secondary eczematous changes
**Congenital immunodeficiencies**			
Hyper-IgE syndrome	Infants	Rare	Pustular and eczematous rashes within first weeks of life; staphylococcal infections of the skin, sinuses, and lungs; high serum IgE; eosinophilia
Wiskott-Aldrich syndrome	Infants	Very rare	Rash identical to that of AD, usually in first weeks of life in boys; microthrombocytopenia
Omenn syndrome	Infants	Very rare	Early-onset erythroderma, diffuse scaly rash, and chronic diarrhea
**Keratinization disorders**			
Ichthyosis vulgaris	Infants and adults	Uncommon	Dry skin with fine scaling, particularly on the lower abdomen and extensor areas; perifollicular skin roughening; palmar hyperlinearity; full form (i.e., 2 *FLG* mutations) is uncommon; often coexists with AD
Netherton syndrome	Infants and adults	Very rare	Eczematous lesions spread over the skin in a serpiginous linear pattern with double-edged scales; hair shaft anomalies (bamboo hair); increased IgE; eosinophilia
**Nutritional deficiency**			
Zinc deficiency	Children	Uncommon	Erythematous scaly patches and plaques most often around the mouth and anus; rare congenital form accompanied by diarrhea and alopecia
**Neoplastic disease**			
Cutaneous T-cell lymphoma	Adults	Uncommon	Erythematous pink-brown macules and plaques with a fi ne scale; poorly responsive to topical steroids; variable itch (in early stages)

*FLG*: filaggrin; AD: atopic dermatitis

*Common = roughly 1 in 10 to 1 in 100; uncommon = roughly 1 in 100 to 1 in 1000; rare = roughly 1 in 1000 to 1 in 10 000; very rare = less than 1 in 10 000

†Especially in developing countries

Adapted from: Weidinger S, Novak N. Lancet. 2016;387(10023):1109-22 [12]

## Allergy assessment

The exact role of foods and aeroallergens in the pathogenesis and exacerbation of AD is controversial. Although most patients with AD demonstrate specific IgE antibodies to foods and/or aeroallergens on skin prick testing (SPT) and measurements of serum-specific IgE levels, their clinical significance remains unclear [32, 37]. While a positive SPT or serum-specific IgE test indicates sensitization to a particular allergen, this does not prove clinical hypersensitivity or causation.

For the vast majority of children with AD, foods are not a trigger. In clinical studies, as many as 35% of children with moderate-to-severe AD have clinically relevant food allergies [37], however these are most often associated with immediate symptoms upon ingestion, and are usually clearly identifiable from the clinical history [10]. In contrast, food allergies appear to have little, if any, role in adult AD [32].

Random testing or screening to food allergens in patients with AD is not recommended as this may lead to unnecessary and inappropriate dietary restrictions. The positive predictive value of screening panels of food allergens in such cases is as low as 2%, and these screening panels are associated with significant health care utilization [38]. Unnecessary dietary restrictions can result in nutritional deficiencies, adversely impact child development (e.g., food aversions and abnormal eating habits) and can paradoxically increase the risk for developing immediate and potentially life-threatening food allergies for some patients. Therefore, the decision to perform allergy testing to foods should be based on whether or not the patient’s history is consistent with or highly suggestive of IgE-mediated food allergies [37]. Evidence suggests that strict elimination diets in children with food-triggered AD should be prescribed with caution as they can inadvertently lead to loss of tolerance of foods and increase the risk of immediate, potentially life-threatening IgE-mediated food reactions [39].

Exposure to aeroallergens such as house dust mites, animal dander, pollen and moulds can exacerbate AD in some patients. In these cases, identification of sensitization by SPT may be useful. Specific avoidance measures should be considered since removal of the allergen from the patient’s environment may improve the symptoms of AD. Atopy patch testing is still considered investigational in patients with AD because there are no standardized methods of application or test interpretation. However, patch tests may be useful for excluding a diagnosis of concurrent contact dermatitis [32].

## Prevention

Although there are currently no established primary prevention strategies for AD, recent trials have demonstrated the effectiveness of early, consistent application of emollients for infants at increased risk. Probiotic use has also been intensively studied.

### Emollients

This simple and cost-effective approach has resulted in a 30–50% reduction in the diagnosis of AD at 6–12 months in some studies [40–43]. A meta-analysis suggested that the benefit of emollients is only seen in studies that included high-risk infants and when emollient application was continued throughout the study duration [44]. This implies emollients may perhaps be delaying, rather than preventing, disease expression and that continuous use is required for benefit.

A recent randomized study found no significant difference in effectiveness between the main types of emollients used for childhood AD (i.e., lotions, ointments, gels, creams) [45]. Therefore, the ideal option is the one that the patient tolerates best and is most likely to adhere to.

Early-onset, severe AD is the strongest risk factor for the development of food allergy in children [46] and, hence, it has been specultated that the prevention of AD may prevent food allergy. The dual allergen exposure hypothesis proposes that there is a narrow window of opportunity in the first year of life where allergen exposure, first through the skin, as opposed to the gut, leads to food allergy. Based on this hypothesis, trials have been undertaken to look at whether early emollient use can prevent food allergy. Both meta-analyses and subsequent large randomized controlled trials have failed to demonstrate the efficacy of early emollient use for food allergy prevention [43, 44, 47, 48].

### Probiotics

Patients with AD have gut microbial alterations showing increased *Escherichia coli*,* Clostridium difficile* and *Staphylococcus aureus*, and a decrease in beneficial microbes, such as *Lactobacillus* and *Bifidobacterium* [49]. In the first year of life, the gut microbiome changes rapidly, and it is enriched by the interaction with the external environment. From the second year of life and beyond, the child’s gut microbiome begins to stabilize and increasingly resembles that of an adult, reaching an adult-like microbiome profile in later childhood [50].

The Food and Agriculture Organization of the United Nations (FAO) and the World Health Organization (WHO) have defined probiotics as “live microorganisms, which when administered in adequate amounts confer a health benefit on the host.” [51]. Oral probiotic supplementation, a frequently studied microbiome-based intervention, provides a mechanism to change the postnatal gut microbiome. Two recent systematic reviews and meta-analyses demonstrated a 30–40% reduction in the incidence of AD with probiotic use compared to controls [52, 53]. Sub-analyses in both meta-analyses found that inclusion of maternal probiotics was essential for benefit, but findings for infant probiotic use were discordant. Since studies on probiotics have been complicated by considerable differences in microbial composition, dose, timing, and clinical trial designs, specific recommendations for their use in the prevention of AD cannot be made and await further research.

## Treatment

The treatment of AD should be directed at restoring the skin barrier, which includes hydrating and repairing the skin, limiting itching, and decreasing inflammation when necessary. Therefore, the successful management of AD requires a multifaceted approach that involves patient and caregiver education, optimal skin care practices, anti-inflammatory treatment with topical corticosteroids (first-line), topical calcineurin inhibitors (TCIs) and/or phosphodiesterase-4 (PDE-4) inhibitors as well as the treatment of skin infections [1, 11, 12, 32]. Systemic immunosuppressive agents, including the recently approved selective JAK inhibitors, may also be considered in severe cases that cannot be controlled with appropriate skin care and topical therapy. We have entered a new era of biologic therapies for AD which have comparable efficacy to other systemic agents with less need for monitoring and fewer potential safety concerns. Second-generation antihistamines can be considered as an adjunct to reduce pruritis which is often the most bothersome symptom for patients [54]. The use of first-generation antihistamines is discouraged due to their unfavourable side-effect profile [55].

**Fig. 4:**
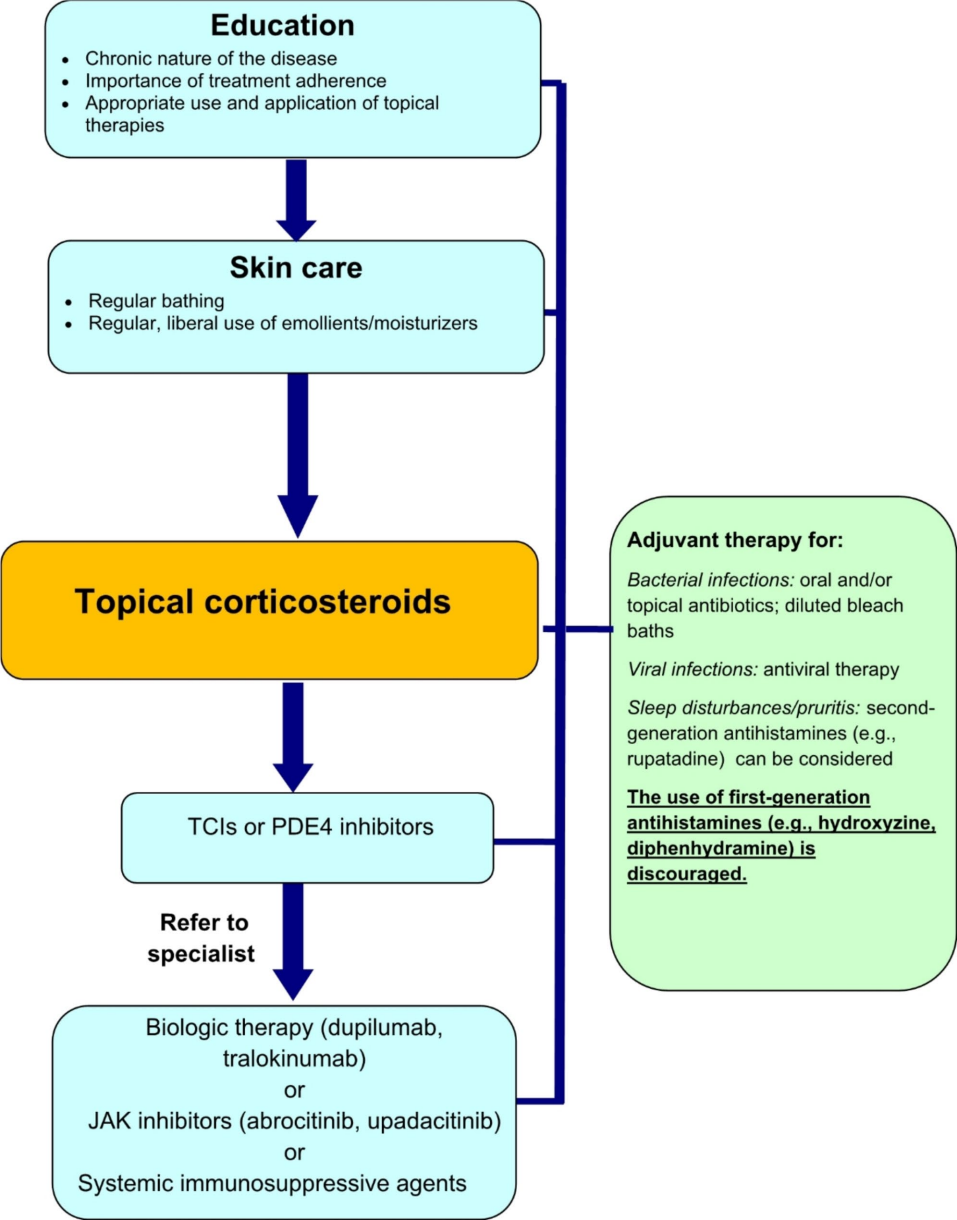
A simplified, stepwise algorithm for the treatment of AD. TCIs: topical calcineurin inhibitors; PDE4: phosphodiesterase-4; JAK: Janus Kinase

A simplified, stepwise algorithm for the treatment of AD is provided in Fig. 4. Physicians should monitor patient progress and disease course regularly and evaluate the efficacy and tolerability of therapy. Follow-up evaluations should include an assessment of medication use (e.g., type, quantity applied, refills made, etc.), which allows the physician to gauge compliance and medication risks. Referral to a specialist (allergist or dermatologist) should be considered for those patients with severe flares or persistent disease despite appropriate use and adherence to topical therapies. In these cases, the specialist may consider more advanced therapies, such as biologics or JAK inhibitors. Referral to an allergist should also be considered for those patients or families worried about the potential role of allergies and for those who also have significant allergic co-morbidities, such as food allergy or asthma.

### Education

For optimal disease management, patients and/or their caregivers should be educated about the chronic nature of the disease, the need for continued adherence to proper skin care practices, and the appropriate use and application of topical therapies. Poor treatment outcomes are often related to poor adherence, especially to topical therapies, resulting from irrational fears about adverse effects and insufficient information [12]. Time spent addressing these fears and educating patients and caregivers has been shown to have a positive effect on disease outcomes. Patients should also be provided with written instructions/information on appropriate medication use, skin care and flare management to reinforce learning, and the choice of which agent to use should be made via shared-decision making. Emphasis should be made on improving symptoms and QoL and highlighting the paucity of data suggesting any treatment leads to cure. Counselling is paramount as many patients may have received misguided information that specific food or environmental allergens are the cause of their eczema and discussions are required to set realistic patient expectations and goals of care.

### Skin care principles

A key feature of AD management is appropriate daily skin care. Although the frequency of bathing is somewhat controversial, most experts suggest daily bathing [56]. A randomized trial found an improvement in eczema severity with twice-daily bathing compared to twice-weekly bathing [57]. Bathing once or twice daily (depending on the severity of AD) in warm water for 10–15 min is recommended to help hydrate and cleanse the skin, assist in the debridement of infected skin, and improve the penetration of topical therapies. Moisturizing cleansers are recommended while highly fragranced soaps should be avoided as they may irritate the skin. After bathing, the patient’s skin should be patted dry with a towel (so it remains slightly wet) and moisturizers/emollients should be applied liberally to help prevent moisture loss and drying of the skin. Experts recommend that patients purchase inexpensive brands of creams or ointments that are available in large containers/jars.

### Topical corticosteroids

Topical corticosteroids are the first-line pharmacologic treatments for AD. These agents effectively control atopic flares through their anti-inflammatory, antiproliferative, and immunosuppressive actions. Numerous topical corticosteroids are available in Canada, ranging from low to high potency, and most of these agents are available in varying concentrations, preparations and doses (see Table 4). Topical corticosteroids are applied to the red, itchy or inflamed areas on the skin before the use of emollients. Some patients have inadvertently reversed the order, which significantly reduces the benefits of the topical corticosteroid.

**Table 4:** Potency of common topical corticosteroid therapies

**Very potent:**	**Moderately potent:**
· Betamethasone dipropionate (Diprolene) · Clobetasol propionate 0.05% (Dermovate) · Halobetasol propionate (Ultravate) · Halcinonide 0.1% (Halog) **Potent**: · Amcinonide 0.1% (Cyclocort) · Betamethasone valerate 0.1% (Betaderm, Celestoderm, Prevex) · Desoximetasone 0.25% (Desoxi, Topicort) · Diflucortolone valerate 0.1% (Nerisone) · Fluocinolone acetonide 0.25% (Derma, Fluoderm, Synalar) · Fluocinonide 0.05% (Lidemol, Lidex, Tiamol, Topsyn) · Fluticasone propionate (Cutivate) · Mometasone furoate 0.1% (Elocon)	· Betamethasone valerate 0.05% (Betnovate) · Betamethasone valerate 0.05% (Celestoderm) · Clobetasone butyrate 0.05% (Eumovate) · Hydrocortisone valerate 0.2% (Westcort, HydroVal) · Prednicarbate 0.1% (Dermatop) · Triamcinolone acetonide 0.1% (Aristocort R, Kenalog, Traiderm) **Mild**: · Desonide (Desocort) · Hydrocortisone acetate 0.5-2% (Cortef, Hyderm, Cortate, Cortoderm)

There is limited clinical trial data to assist in choosing a corticosteroid. Ointment preparations are generally preferred over creams as they provide more uniform coverage and penetration. Also, the least potent preparation required to control AD (particularly in sensitive areas such as the face, neck, groin, and underarms) should be utilized. Often, a low-potency preparation, such as hydrocortisone acetate 1% or equivalent, is used for the face.

When used appropriately, topical corticosteroids are extremely safe and effective. Possible local side effects of long-term topical corticosteroid use include striae (stretch marks), petechiae (small red/purple spots), telangiectasia (small, dilated blood vessels on the surface of the skin), skin thinning, atrophy and acne; however, these effects are uncommon with low or moderate potency preparations. Systemic side effects with topical corticosteroid use are rare, and are usually associated with higher-potency preparations being applied to a large body surface area.

Evidence also suggests that topical corticosteroids may be beneficial for the prophylaxis of AD flares. Studies have found that, after AD is stabilized, the addition of twice-weekly topical anti-inflammatories to maintenance treatment with emollients significantly reduces the risk of relapses in both pediatric and adult subjects [58].

### Topical calcineurin inhibitors (TCIs)

TCIs are immunosuppressant agents that have also been shown to be safe and effective for the treatment of AD [12, 59, 60], as well as the prophylaxis of AD flares [58]. Two TCIs — pimecrolimus (Elidel) and tacrolimus (Protopic) — are currently approved in Canada for the second-line, intermittent treatment of immunocompetent patients with moderate-to-severe AD [61, 62]. Pimecrolimus is approved for patients 3 months of age and older and tacrolimus is approved for those 2 years of age and older. Twice-weekly tacrolimus has been shown to prevent the frequency of flareups in both pediatric and adult patients and can be considered for maintenance therapy without the safety concerns that limit topical corticosteroid use [63].

The most common local adverse effects of TCIs are skin burning and irritation, which often improve with continued use. Although there was initially some concern about an increased risk of malignanices with TCIs, current data have refuted this [64, 65]. The Canadian Dermatology Association has updated their position statement to conclude that there continues to be no evidence of increased malignancy rates in adult and pediatric patients treated with these agents [66–69] and counselling may be required to reassure patients of this [70].

Given the high costs of TCIs, they are generally reserved for patients with persistent disease and/or frequent flares that would require continuous topical corticosteroid treatment, or in patients severely affected in sensitive skin areas (e.g., around the eyes, face, neck and genitals) where systemic absorption and the risk of skin atrophy with topical corticosteroids are of particular concern.

### Phosphodiesterase-4 (PDE-4) inhibitors

PDE-4 inhibitor therapy (e.g., crisaborole 2% ointment) is an alternative option to topical corticosteroids and TCIs for mild to moderate AD. Crisaborole ointment (Eucrisa) is currently approved in Canada for patients 3 months of age or older with mild to moderate AD [71]. In a clinical study, crisaborole ointment improved skin and pruritis scores in children and adults with AD and had a favourable safety profile [72]. Long-term safety data for crisaborole ointment are also reassuring; the most common side effects are application site pain, discomfort or erythema [73]; patients should be counselled on these potential side effects. Less commonly, application site infection can occur [74]. Note that, like TCIs, PDE-4 inhibitors are more expensive than most topical corticosteroids which may limit their use in those without public or private coverage.

### Treatment of skin infections

The skin of patients with AD is often heavily colonized with *S. aureus*, even at uninvolved sites. Short-term topical and/or oral antibiotic therapy is recommended when an overt secondary bacterial infection is present. Appropriate systemic antibiotics are indicated for widespread secondary infection, and first- or second-generation cephalosporins or anti-staphylococcal penicillins for 7 to 10 days are usually effective in managing the infection. Because erythromycin-resistant organisms are common in patients with AD, macrolides are less useful alternatives [32].

Patients with AD are also prone to recurrent viral infections. Eczema herpeticum (a severe disseminated herpes infection that generally occurs at sites of skin damage; also known as Kaposi’s varicelliform eruption) is a serious risk in patients with widespread AD and may be easily misdiagnosed as a bacterial superinfection. Patients with this condition will require systemic antiviral treatment with acyclovir or other antiviral agents [32]. Molluscum contagiosum (a common viral cutaneous infection caused by a poxvirus of the *Molluscipox* genus) is often seen in children with AD. Although the infection is usually self-limited, the lesions often resolve slowly and tend to spread in patients with AD [12]. Severe, persistent molluscum contagiosum infection may require laser and/or antiviral therapy.

Diluted bleach baths are also recommended to help reduce the number of *S. aureus* skin infections, and the need for systemic antibiotics in patients with heavily colonized skin. Diluted bleach baths involve soaking the patient for approximately 10 min in a tub full of lukewarm water that is mixed with one-quarter to one-half cup (60–120 mL) of chlorine bleach (this concentration is similar to the amount of chlorine in a pool). The patient is then thoroughly rinsed with fresh water, and a moisturizer or emollient is applied immediately to prevent dehydration and dryness [1]. A recent systematic review and meta-analysis provided moderate quality evidence that two to three times weekly bleach baths reduced eczema severity, with little to no difference in adverse outcomes compared to no bleach baths [75].

### Biologic agents

In the last few years, two biologic agents have been approved in Canada for moderate to severe AD not responding to topical therapies. Dupilumab (Dupixent) was the first biologic to be approved in adults, and now has approval for patients 6 months of age and older [76]. Tralokinumab (Adtralza) is approved for patients 12 years of age and older [77]. Dupilumab is an interleukin (IL)-4 receptor alpha antagonist which leads to inhibition of IL-4 and IL-13 signaling. Tralokinumab binds to and neutralizes the effects of IL-13. These cytokines play an integral role in the hyperactive T2 inflammatory cascade found in patients with eczema, and blocking them significantly reduces this pathway’s activity. Dupilumab and tralokinumab are injection therapies that can be self-administered via pre-filled pen or syringe, depending on the patient’s age. Both biologics have been shown to significantly improve eczema scores (Eczema Area and Severity Index [EASI], Investigator’s Global Assessment [IGA] and others), pruritis scores and QoL scores when used as monotherapy or concomitantly with topical corticosteroids [78–95]. No head-to-head trials have been performed to compare these two biologic agents. It is important to note that, as with all agents approved for AD, these biologics do not lead to remission of eczema and, therefore, symptoms will recur when these therapies are discontinued.

Short- and long-term safety data have been published for both dupilumab and tralokinumab [78–95]. Injection-site erythema and/or pain are the most common side effects. Conjunctivitis is a more significant side effect, and occurs more frequently with dupilumab than tralokinumab [96]. Patients should seek medical attention if this side effect occurs, and referral to ophthalmology may be required. Needle-phobic patients may find dupilumab and tralokinumab less desirable options, however, it is important to note that, unlike other systemic agents, these biologics do not require bloodwork monitoring at initial work-up and following treatment initiation.

### Systemic immunosuppressive agents

Short-term treatment with systemic immunosuppressive agents, such as cyclosporine, azathioprine and methotrexate, has been shown to be effective in patients failing topical treatment and, until late 2017, were the only agents available to treat severe, refractory AD [11, 12]. These older systemic agents may still need to be considered in some cases despite advances in treatment with safer alternatives, such as biologics and JAK inhibitors, due to the high costs of these newer options. It is important to note that discontinuation of these immunosuppressives, in particular cyclosporine, often leads to rapid disease relapse. Also, patients treated with these immunosuppressive agents should be monitored for potential adverse effects, such as kidney or liver function impairment with cyclosporine, and myelosuppression with azathioprine. Of note, referral to a specialist should be made for AD patients who may be candidates for systemic therapy.

JAK inhibitors have been in use for several years for rheumatological conditions. In 2021, upadacitinib was the first JAK inhibitor to be approved for moderate-to-severe AD, followed by abrocitinib in 2022. Both of these agents are approved for patients 12 years of age and older [97, 98]. The JAK inhibitors for eczema selectively inhibit the JAK–STAT pathway, thereby significantly reducing pro-inflammatory cytokine activity but sparing the side effects of JAK-2 inhibition (neutropenia and anemia) [99]. Both upadacitinib and abrocitinib have been shown to improve disease severity, pruritis and QoL scores [100–109]. Head-to-head trials of JAK inhibitors and biologics for moderate-to-severe AD have been done and showed comparable benefits [108, 109]. Therefore, the patient and specialist can decide which systemic agent is best through shared-decision making.

Both JAK inhibitors approved for AD are once-daily oral tablets and have low and high doses available [97, 98]. These agents are not recommended in pregnancy and, therefore, female patients of child-bearing age should be on contraception while on treatment. Prior to initiation of JAK inhibitor treatment, patients need to be screened for chronic infections, including tuberculosis and hepatitis. Other laboratory monitoring required at both treatment initiation and during follow-up includes: complete blood counts, lipids and hepatic and renal function. The most common side effects associated with JAK inhibitors are mild and include acne and nausea. However, opportunistic infections, such as herpes zoster and eczema herpeticum, have also occurred. Similar to other immunosuppressive agents, patients should be counselled on the risk of malignancy, thrombosis and serious opportunistic infections, although the risk of these events with the JAK inhibitors used in AD is low.

Systemic corticosteroids have an unfavourable risk–benefit profile, and there is currently insufficient evidence supporting their use in AD. Therefore, these agents should be reserved for exceptional cases, and prolonged use should be avoided given their potential for serious adverse events [12].

### Antihistamines

Although first-generation antihistamines (e.g., hydroxyzine, diphenhydramine, chlorpheniramine) do not directly affect the itching associated with AD, the sedative effects of these agents have been found to help improve sleep in patients with AD [1, 32]. However, given their unfavourable safety profile, which includes reduction in rapid eye movement (REM)-sleep, impaired learning and reduction in work efficiency [110], they should not be routinely recommended. Non-sedating second-generation antihistamines appear to provide modest benefit in AD patients with allergic triggers [1, 32, 54] and, hence, a therapeutic trial of these agents may be considered in certain clinical situations.

### Other therapies

Ultraviolet (UV) phototherapy may be beneficial for the treatment of AD in adults. However, the long-term toxicity of UV therapy is still unknown. Allergen immunotherapy (for allergens other than foods) may also be effective in select patients with AD that is associated with aeroallergen sensitization (see *Allergen Immunotherapy* article in this supplement) [111–113]. A meta-analysis found sublingual and subcutaneous allergen immunotherapy, in particular dust mite immunotherapy, to be associated with significant improvements in AD scores and QoL indicies [114].

Although some studies have found wet-wrap therapy (the application of wet bandages over AD lesions after applying emollients and/or topical corticosteroids) to be effective for the treatment of AD, others have questioned its effectiveness and emphasize the potential for associated complications such as local infections [12]. A systematic review of trials comparing wet wrap therapy to conventional topical corticosteroid treatment in patients with AD found no good quality evidence to suggest that wet wraps are superior to conventional topical therapies [115].

There are many other emerging therapies for AD that are not yet approved in Canada, including topical JAK inhibitors and other topicals modulating the microbiome, and numerous other biologic targets and oral JAK inhibitors. Therefore, the options for severe or refractory patients are expanding extensively.

## Prognosis

The prognosis for patients with AD is generally favourable, with most children going into clinical remission by early adolescence. It is important to counsel parents, however, on the replasing-remitting nature of eczema and the possibility of recurrence in the future. Patients with severe, widespread disease and concomitant atopic conditions, such as asthma and allergic rhinitis, are likely to experience poorer outcomes [25]. Fortunately, we now have a wider range of treatment options for these patients to improve their QoL than we did a few years ago, and many can achieve normal or near normal skin while on treatment.

## Conclusions

AD is a common, chronic skin disease that often starts early in life and can adversely impact the QoL of patients and their caregivers. Optimal skin care practices and topical corticosteroids remain the cornerstone of therapy for the disease. TCIs and PDE-4 inhibitors are effective, second-line alternatives to topical corticosteroids in appropriate patients prone to frequent flare-ups. Newer systemic agents, such as JAK inhibitors and biologics, have expanded the range of available options for severe cases that cannot be controlled with appropriate skin care and topical therapies. However, in some of these severe patients, the older systemic immunosuppressive agents may still need to be trialed. Allergy testing to foods and aeroallergens may be considered based on patient history and/or in patients exhibiting a poor response to optimal skin care practices and appropriate pharmacological therapy.

## Data Availability

Data sharing not applicable to this article as no datasets were generated or analyzed during the development of this review.

## Declarations

### About this supplement

This article has been published as part of Allergy, Asthma & Clinical Immunology, Volume 20 Supplement 03, 2024: Practical Guide for Allergy and Immunology in Canada 2024. The full contents of the supplement are available at https://aacijournal.biomedcentral.com/articles/supplements/volume-20-supplement-3.

## Abbreviations

AD: Atopic dermatitis
FLG: Filaggrin gene
FAO: Food and agriculture organization of the united nations
IL: Interleukin
IgE: Immunoglobulin E
JAK: Janus kinase
PDE-4: Phosphodiesterase-4
QoL: Quality of life
SPT: Skin prick testing
TCIs: Topical calcineurin inhibitors
UV: Ultraviolet
WHO: World health organization
